# Supplementary figures and images for: Epicatechin modulates stress-resistance in C. elegans via insulin/IGF-1 signaling pathway
Source: PLoS One. 2019 Jan 28;14(1):e0199483. doi: 10.1371/journal.pone.0199483 (PMC6349306; doi:10.1371/journal.pone.0199483)

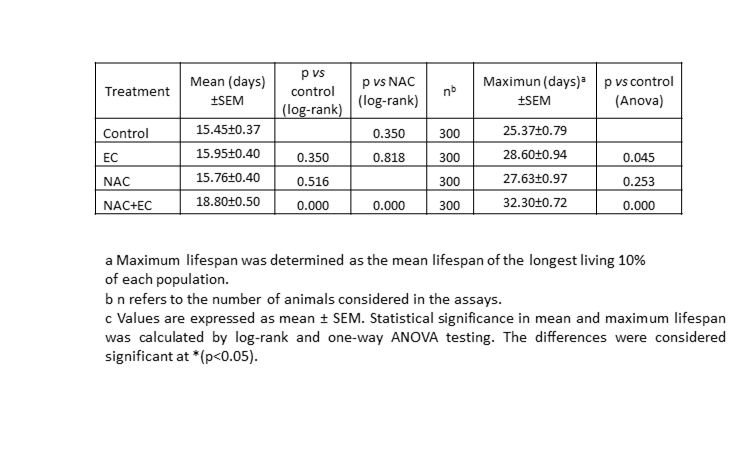

Supplement: S1 Table — (TIF) [file pone.0199483.s001.tif]

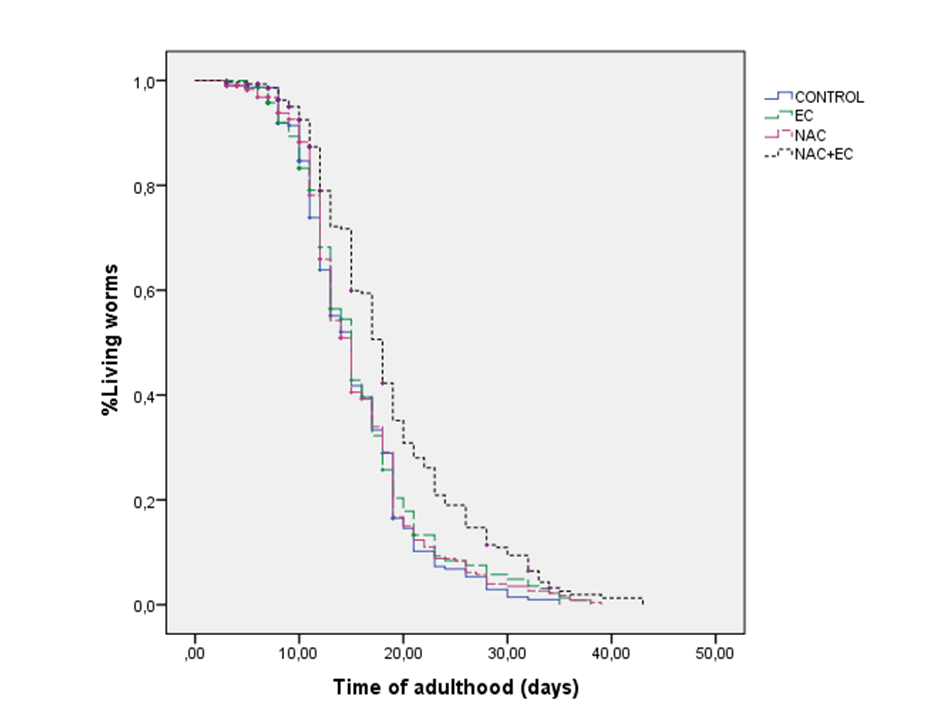

Supplement: S1 Fig — (TIF) [file pone.0199483.s002.tif]

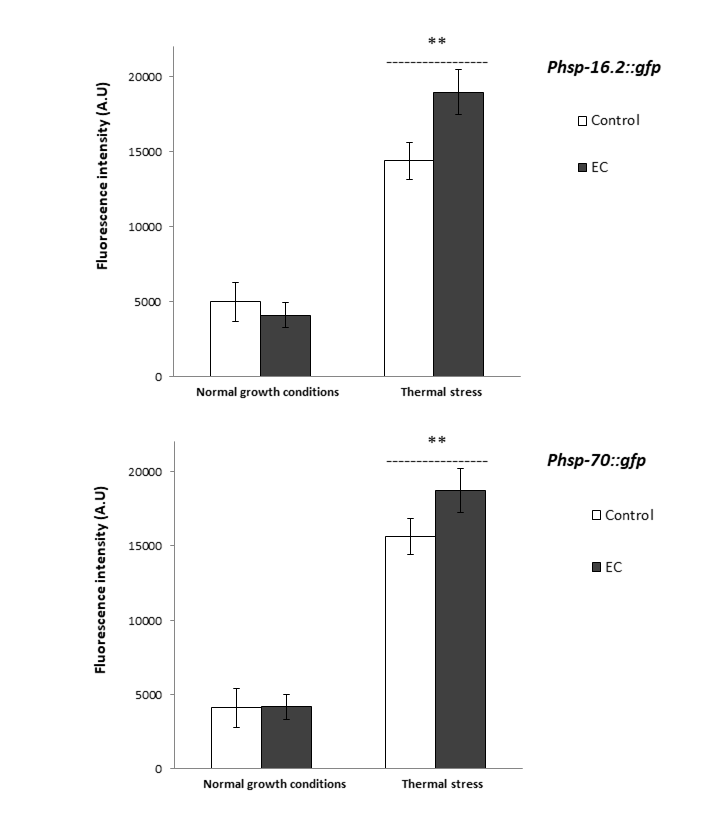

Supplement: S2 Fig — Age- synchronized L1 transgenic worms expressing Phsp-16.2::gfp and Phsp-70::gfp transgenes were cultivated in the absence (controls) and presence of EC (200 μM) in the culture media. Relative GFP fluorescence intensities in transgenic A) Phsp-16.2::gfp and B) Phsp-70::gfp worms were quantified under normal growth conditions and after subjecting worms to thermal stress to 35°C for 1h. Total GFP fluorescence of each whole worm was quantified using Image J software. Three independent experiments were performed. The results are presented as the mean values ± SEM. Approximately 35 randomly selected worms from each set of experiments were examined. Differences compared with the control (0 μM, 0.1% DMSO) were considered statistically significant at p<0.05 (*) and p<0.01 (**) and p<0.001 (***) by one-way ANOVA. (TIF) [file pone.0199483.s003.tif]

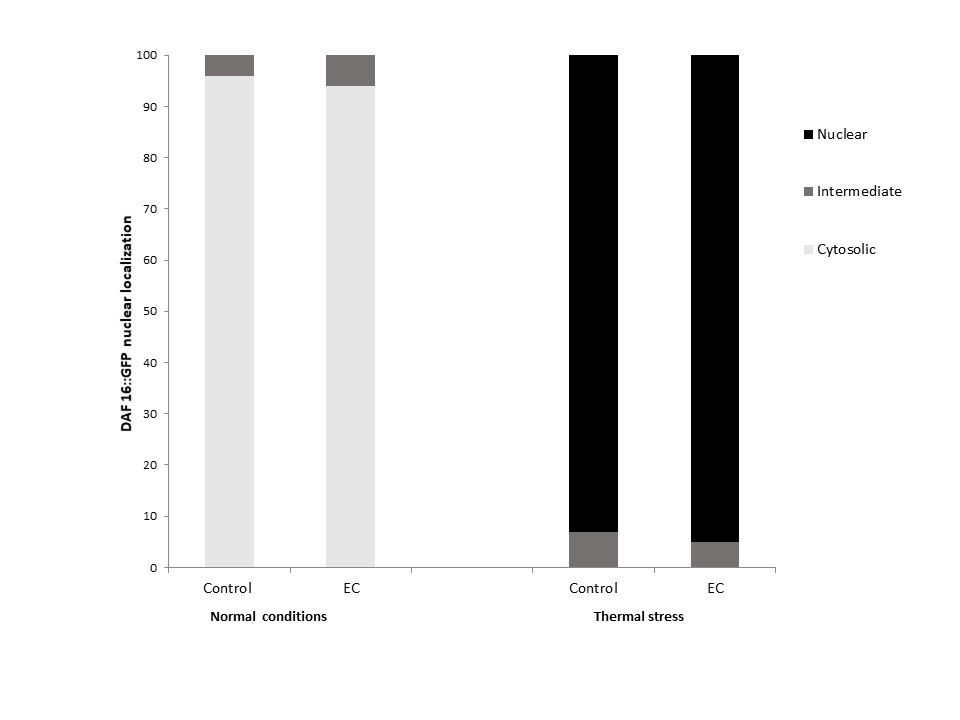

Supplement: S3 Fig — Transgenic worms expressing the DAF-16::GFP fusion protein were cultivated in the absence (controls) and presence of EC (200 μM) and evaluated at 9th day of adulthood. DAF-16:GFP subcellular localization was classified as cytosolic, intermediate and nuclear. (TIF) [file pone.0199483.s004.tif]

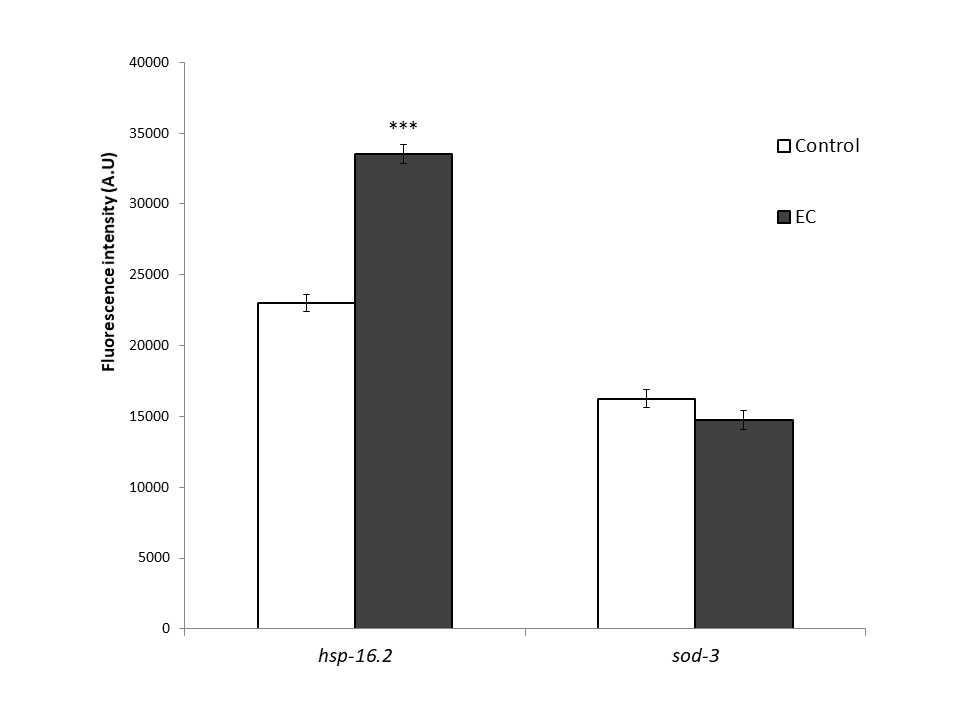

Supplement: S4 Fig — Age-syncronized L1 transgenic worms of Psod-3:gfp and Phsp-16.2:gfp were cultivated in the absence (controls) and presence of EC (200 μM) in the culture media. Total GFP fluorescence of each whole worm was quantified using Image J software. Three independent experiments were performed. The results are presented as the mean values ± SEM. Approximately 35 randomly selected worms from each set of experiments were examined. Differences compared with the control (0 μM, 0.1% DMSO) were considered statistically significant at p<0.05 (*) and p<0.01 (**) and p<0.001 (***) by one-way ANOVA. (TIF) [file pone.0199483.s005.tif]
